# *Limosilactobacillus fermentum* CECT5716: Clinical Potential of a Probiotic Strain Isolated from Human Milk

**DOI:** 10.3390/nu15092207

**Published:** 2023-05-06

**Authors:** Metehan Ozen, Hugues Piloquet, Monika Schaubeck

**Affiliations:** 1 School of Medicine, Acibadem Mehmet Ali Aydinlar University, 34752 Istanbul, Türkiye; metehanozen@yahoo.com; 2 Department of Paediatric Chronic Diseases, Nantes University Hospital, 44000 Nantes, France; hugues.piloquet@chu-nantes.fr; 3 HiPP GmbH & Co. Vertrieb KG, 85276 Pfaffenhofen, Germany

**Keywords:** *Limosilactobacillus fermentum* CECT5716, probiotic, human milk, infant formula, infant microbiota, *Lactobacillus fermentum* CECT5716, respiratory tract infections, diarrhoea

## Abstract

Breastfeeding provides the ideal nutrition for infants. Human milk contains a plethora of functional ingredients which foster the development of the immune system. The human milk microbiota predominantly contributes to this protective effect. This is mediated by various mechanisms, such as an antimicrobial effect, pathogen exclusion and barrier integrity, beneficial effects on the gastrointestinal microbiota, vitamin synthesis, immunity enhancement, secreted probiotic factors, and postbiotic mechanisms. Therefore, human milk is a good source for isolating probiotics for infants who cannot be exclusively breastfed. One such probiotic which was isolated from human milk is *Limosilactobacillus fermentum* CECT5716. In this review, we give an overview of available interventional studies using *Limosilactobacillus fermentum* CECT5716 and summarise preclinical trials in several animal models of different pathologies, which have given first insights into its mechanisms of action. We present several randomised clinical studies, which have been conducted to investigate the clinical efficacy of the *Limosilactobacillus fermentum* CECT5716 strain in supporting the host’s health.

## 1. Introduction

Breastfeeding is the ideal nutrition for infants, providing the neonate with all the required macro- and micronutrients in an individualised and optimal way. Besides nutrients, human milk (HM) contains a plethora of non-nutritive functional ingredients, e.g., immunoglobulins, human milk oligosaccharides (HMO), microorganisms, and cytokines, supporting the neonate´s specific immune system and tolerogenic immune response [1]. The role of HM in preventing infections is well established [2,3,4]. Therefore, exclusive breastfeeding is recommended in the first six months of life [5]. The mechanisms of this protective effect are not only based on direct immunological effects (anti-infectious substances such as immunoglobulins) but also on the modulation of the microbiota, specifically, by the human milk microbiota (HMM) [6].

The microbial community of HM is transferred to the infant during breastfeeding (vertical transmission) and plays an important role in the development of the infant’s intestinal microbiota, immune response, and pathogen defence. These microbes contribute to the health-promoting effects of HM via various mechanisms, e.g., via the production of antimicrobial compounds [7]. Scientific evidence shows that the HMM is an infant-tailored protective entity influenced by several factors: internal factors (e.g., genetic predisposition regarding HMO) and external factors (e.g., delivery mode, perinatal antibiotics) are associated with alterations in the HMM. Correlations have also been observed for the phase of lactation (more lactobacilli in the neonatal phase and more bifidobacteria in mature milk) and sex of the infant [8,9] or mother´s nutrition and geographic location [10].

The presence of staphylococci, streptococci, corynebacteria, propionibacteria, lactobacilli, and bifidobacteria was detected in the vast majority of HM samples tested in different cohorts via different methods, leading to the hypothesis that these are part of the core microbiota, i.e., the microbial taxa most often found to be part of the HMM in different studies [1,11]. Recent estimations suggest that HM contains approximately 10^3^ colony forming units (CFU) per ml. Previous estimations were higher, possibly due to less stringent disinfection of surrounding skin [7]. Although most studies on the HMM focus on bacteria, some studies detected fungi in HM [12]. Furthermore, a virome unique to HM was identified that may be transmitted to the infant, which will be very interesting for future studies to investigate [13].

While the origin of the HMM is not yet fully understood, several routes have been hypothesised: (1) contamination from skin microbiota, (2) engraftment of microbes from the infant´s mouth via retrograde flow, and (3) translocation of gut-resident microbes, via the entero-mammary pathway. All three factors might synergise in generating a diverse health-promoting HMM.

The isolation of probiotic strains from HM may present a possibility to transfer the HMM-mediated protection to infants who cannot be breastfed [6]. As probiotics are assumed to be host specific, the characterisation of probiotic candidates from human sources is a way to obtain effective probiotic strains with health-promoting properties. Isolation from non-human sources might be less effective, due to the inability of engraftment in the intestinal microbiota [14]. Therefore, the isolation of new strains from HM or from infant faecal sources is considered the ideal approach to obtain probiotic strains for infants’ health [6]. For instance, lactobacilli are regularly found in the HMM [11], and bifidobacteria are abundant in faeces [10].

Per the definition of probiotics, delivery of the microorganism in its viable form and survival in the gastrointestinal tract (GIT) are required. This ensures that the metabolically active microorganisms produce proactive compounds in the final product or in the gut. In addition to probiotics, HM also contains prebiotics that support the establishment of a characteristic infant microbiota, rich in lactobacilli and bifidobacteria. The aim of microbiota modulation is to foster a healthy microbiota mimicking the microbiota of breastfed infants by using probiotics, prebiotics, or a combination of both (synbiotics). Recently, the term postbiotics emerged after it was shown that some of the protective effects of probiotics can also be observed in a non-viable state [15]. Thus, some studies on probiotics, where inactivated forms of bacterial strains were used, describe postbiotic effects.

*Limosilactobacillus fermentum* (*L. fermentum*) CECT5716, isolated from HM, is a well-known strain in the fields of nutritional research and consumer health. This interest arises due to the actions of *L. fermentum* CECT5716 on the immune system and intestinal barrier. Health-promoting effects have been shown in vitro, in vivo, and in human studies. In this review, we describe the mechanisms by which the probiotic strain *L. fermentum* CECT5716 confers health-promoting effects. We further summarise its safety profile and the results of relevant preclinical and clinical studies with *L. fermentum* CECT5716.

## 2. *L. fermentum* CECT5716

The genus of *Lactobacillus* has grown very large and diverse. Therefore, the taxonomy has recently been updated, thus introducing new genus names. Based on physiology and genomics, *Lactobacillus fermentum* was therefore re-categorised as *Limosilactobacillus fermentum* [16]. It still remains within the family of *Lactobacillaceae* (order: *Lactobacillales*; class: *Bacilli*; phylum: *Bacillota* (formerly *Firmicutes*)).

The presence of the *L. fermentum* species in HM was confirmed in several cohorts and studies performed by various research groups worldwide [7,8,17]. *L. fermentum* is a normal inhabitant of the human GIT, including that of breastfed infants [18,19,20]. The *L fermentum* CECT5716 strain was categorised originally as a strain within the species of *Lactobacilli*, which contains Gram-positive, catalase-negative, non-sporulating, rod-shaped organisms. The abundance of lactobacilli in adults is lower than in infants and seems to be even lower in industrialised countries and in unfavourable dietary habits [21].

Lactobacilli in HM were first discovered in 2003, by analysing bacteria from different habitats of healthy mother–infant pairs during physiological lactation. This discovery prompted the hypothesis that the HMM could have probiotic benefits for the infant [22].

After testing different isolated strains from HM, one strain in particular, catalogued as *L. fermentum* CECT5716, displayed superior probiotic properties compared to other strains (e.g., GIT survival) [23]. Therefore, this strain was chosen for further investigation in preclinical and clinical studies, as described in Section 5 and Section 6.

As the benefits of probiotic bacteria are strain specific, a better knowledge of the genetic basis of the strain-specific traits was required. Therefore, the whole genome of *L. fermentum* CECT5716 was sequenced and related to phenotypic traits [24,25], and the fermentation profile of *L. fermentum* CECT5716 was established [23].

## 3. Probiotic Properties and Mechanisms of Action of *L. fermentum* CECT5716

In 2001, an expert meeting of international scientists working on behalf of the Food and Agriculture Organization of the United Nations (FAO) and the WHO discussed the emerging field of probiotics and re-defined the term “probiotics” as “live microorganisms which when administered in adequate amounts confer a health benefit on the host” [26,27]. While the probiotic strain has to be in a viable state throughout the shelf-life in the respective product, it also has to survive the harsh conditions of the GIT. Notably, it was shown that even after 80 min and a low pH of 1.8, *L. fermentum* CECT5716 displayed 30% viability and thereby exceeded the viability of some other tested strains [23]. Survival conditions in the infant stomach are expected to be less harsh; therefore, the percentage surviving in the infant stomach is estimated to be higher.

**Figure 1:**
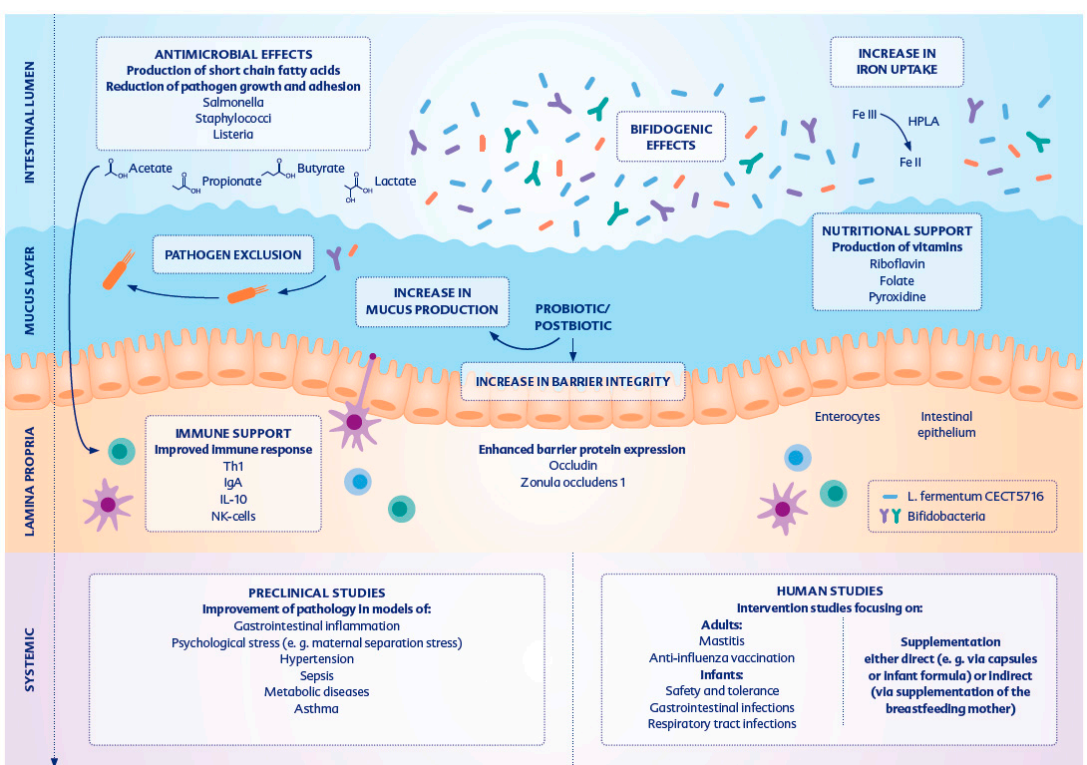
Probiotic and postbiotic mechanisms of *L. fermentum* CECT5716. Probiotic effects in the intestinal lumen were seen for antimicrobial effects [23,29,30], bifidogenic effects [31,32], and nutritional support, as seen by improved iron uptake and vitamin synthesis [24,33]. Positive effects in the mucus layer and direct vicinity of epithelial cells were seen by pathogen exclusion and increased mucus production [23,29,34]. Further effects were seen for an increased epithelial barrier integrity [31,35] and support of the immune system [36,37]. Probiotic and postbiotic effects were seen in preclinical models and in human trials in infants and adults (as detailed in Section 5 and Section 6). IgA: Immunoglobulin A; IL-10: Interleukin 10; HPLA: p-hydroxyphenyllactic acid; Th1: T-helper type 1; NK-cells: Natural killer cells.

Furthermore, prebiotics can support probiotic effects. In vitro, *L. fermentum* reached higher growth levels when supplemented with prebiotics, indicating that certain prebiotic factors contained in HM may foster probiotic growth in the colon [28]. Probiotic properties are strain dependent, and therefore, probiotic candidates are not chosen solely on their capability to survive in the GIT, as this trait is no indication of true health-promoting capacities. This choice is also based on their ability to inhibit pathogens via mechanisms presented in the following sections. A summary of shown probiotic mechanisms for *L. fermentum* CECT5716 is presented in Figure 1.

### 3.1. Antimicrobial Effect

To prevent pathogens from infecting the host, antimicrobial defence by host cells and commensal microbiota is exerted in close proximity to the intestinal epithelial layer, as the first line of defence. Infants fed HM exhibit significantly lower rates of infectious diseases compared to formula-fed infants [3]. Thus, it is hypothesised that probiotics in the HMM may contribute to antimicrobial defence by inhibition of pathogen growth or adhesion. Inhibitory effects of *L. fermentum* CECT5716 are assumed to be mediated by the production of short-chain fatty acids (SCFA) and lactate [23]. *L. fermentum* CECT5716 inhibited *Salmonella choleraesuis* CECT4155 in vitro and in vivo and repressed the growth of *Staphylococcus aureus*, *Listeria* spp., and *Escherichia coli* in vitro [29]. The antimicrobial effect against *Staphylococcus aureus* was also shown in mothers consuming *L. fermentum* CECT5716 [30]. Moreover, the authors of the present review have confirmed a strong antimicrobial activity of *L. fermentum* CECT5716 in recent experiments, where the highest inhibition was observed against *Serratia marcescens*, *Streptococcus pyogenes*, and *Klebsiella pneumonia* (internal data).

### 3.2. Pathogen Exclusion and Barrier Integrity

The adhesion of probiotics to intestinal epithelial cells is assumed to be an important probiotic trait. The rationale lies within the assumed importance of the close proximity between the probiotic and the epithelium to exert a direct effect on the host cell, e.g., by increasing the amount of mucin [34], or to displace or exclude pathogens from attachment to epithelial cells. *L. fermentum* CECT5716 displayed superior adhesion ability in vitro to HT-29 as well as Caco-2 cells [23]. In addition, increased mucin expression was demonstrated [29].

Another potential mechanism could be the strengthening of the host´s intestinal barrier function. This effect could be mediated by increasing the expression of barrier proteins such as occludin or zonula occludens 1 (ZO-1). It was shown that *L. fermentum* CECT5716 prevented the stress-induced loss of intestinal barrier proteins in rats [35]. This observation was confirmed in a rat model of vascular oxidative stress [31]. However, the mechanisms behind this *L. fermentum* CECT5716-induced protection from barrier loss are yet to be investigated and confirmed in humans.

### 3.3. Effects on the Gastrointestinal Microbiota

The growth promotion of bifidobacteria is referred to as the “bifidogenic effect” and is associated with a healthy faecal microbiota in infants. Exclusively breastfed infants have high a bifidobacteria abundance in their faeces, despite the fact that the HMM is not dominated by bifidobacteria [11,38]. Supplementation of infant formula with bifidobacteria is not always successful in increasing the abundance of bifidobacteria in the infant gut [39]. Therefore, synbiotic factors, i.e., factors that positively influence the abundance of beneficial bacteria, are assumed to be of greater importance to the bifidogenic effect of HM.

One strategy to increase the abundance of bifidobacteria is the addition of prebiotics to infant formula [40]. Another possibility is the addition of probiotics that enable engraftment and support the growth of bifidobacteria. Especially in the neonatal gut, aerotolerant lactobacilli, which could originate from either the HMM or the mother´s vaginal microbiota, play an important role in setting the stage for the colonisation of the strictly anaerobic bifidobacteria. It is hypothesised that lactobacilli reduce intestinal oxygen and thereby might promote the engraftment of bifidobacteria [41]. It was shown in vitro and in vivo that *L. fermentum* CECT5716 has a bifidogenic effect [31,32].

In addition to increasing the abundance of bifidobacteria, other positive effects on the overall composition of microbiota were observed for *L. fermentum* CECT5716, e.g., by increasing the abundance of microbes which produce favourable metabolites or by the reduction in intestinal dysbiosis [42]. In a pig model of formula feeding, a beneficial influence on the faecal microbial composition was demonstrated. In this model, *L. fermentum* CECT5716 was only administered during the suckling phase, and the pigs were subsequently challenged with a high-energy diet during their weaning phase. The addition of *L. fermentum* CECT5716 had positive long-term effects on bacterial SCFAs production (especially propionate) and caecal glucagon-like peptide 1 (GLP-1) secretion [43]. GLP-1 has an important role in various metabolic processes, such as insulin secretion and inhibition of gastric emptying and food intake [44]. Interestingly, this *L. fermentum* CECT5716-induced increase in GLP-1 has been previously observed in a rat model under a high fructose diet challenge [45]. This indicates that probiotic intervention during early phases might have long-term influences on the microbiota composition and host metabolism.

### 3.4. Effects on Vitamin Synthesis

Although most lactic acid bacteria are auxotrophic for several vitamins, certain strains have the ability to synthesise water-soluble vitamins such as those categorised as B-group vitamins (e.g., folate (B9), riboflavin (B2), and cobalamin (B12)) [46]. *L. fermentum* CECT5716 produces riboflavin, pyroxidine (B6), and folate in vitro [24]. Folate is particularly interesting due to high folate requirements in the rapidly growing infant. Further, the gut epithelium has a high turnover and is therefore also dependent on folate in the adult state. In particular, the colonic epithelium might benefit from microbially produced folate [47]. However, it is not clear which genes are involved in the synthesis and whether the amount of vitamins produced indeed contributes to the host´s folate status. Few strains have been identified to contribute to the host’s vitamin status [48]. An improvement in the folate status was demonstrated in vivo and suggests that the bifidogenic effect of HM might mediate this improvement [49].

### 3.5. Effects on Immune Response

Another mechanism by which probiotics mediate their health-promoting effect is via activation of the host’s immune response. In a human trial of anti-influenza vaccination, *L. fermentum* CECT5716 increased the immunomodulatory response upon vaccination, as discussed in more detail in Section 6.3 [50]. Further, an increased interferon-γ (IFN-γ) response was observed in another study [35]. For *L. fermentum* CECT5716, a predominant T-helper type 1 (Th1)- and immunoglobulin A (IgA)-driven immune response was observed, compared to other strains [36].

Interestingly, there are indications that the activation of certain immune response characteristics might be linked to the origin of the strain, thereby emphasising the host specificity of probiotics. Compared to saliva-derived strains, the HM-derived strain *L. fermentum* CECT5716 was demonstrated to induce IL-10 to a larger extent. Further, *L. fermentum* CECT5716 also preferentially activated CD56^+^CD8^+^ natural killer (NK) cells [37], which are assumed to have greater cytotoxic efficacy than their CD8- counterparts [51]. The overall immunomodulatory response, however, was similar when comparing the strains of different origin [37]. As NK cells seem to be important in neonatal immune defence, i.e., before acquired immunity is fully developed, the enhancement of NK cell maturation by the HMM could be of pivotal importance in the neonatal gut, where more NK cells are found than in adults [51,52].

### 3.6. Secreted Probiotic Factors

Compounds secreted by metabolically active microorganisms include protein- and carbohydrate-degrading enzymes, antimicrobial factors, or immune-modulatory factors (e.g., lactocepin, reuterin, or SCFAs) [53,54,55]. By inducing antioxidants in the GIT, probiotics can reduce intestinal inflammation. Antioxidant activity is perceived as an important probiotic characteristic and might be mediated in part by the tripeptide glutathione (gamma-L-glutamyl-L-cysteinyl-glycine)—the major component of the endogenous non-protein sulfhydryl pool [56]. Glutathione neutralises reactive oxygen species and is therefore an important antioxidant in the maintenance of intestinal barrier integrity and prevention of inflammation. Low glutathione levels in humans are connected with several disease states such as cancer, AIDS, Alzheimer’s disease, and Parkinson’s disease [57]. Of note, secretion of glutathione is not a common feature among lactic acid bacteria, but has been shown for *L. fermentum* ME-3 [58,59,60]. *L. fermentum* CECT5716 increases the extracellular glutathione levels by recycling (i.e., oxidation) of reduced glutathione (GSSG) [58,59]. In addition to glutathione, the antioxidant dipeptide ɤ-Glu-Cys is also released by *L. fermentum* CECT5716. The dipeptide ɤ-Glu-Cys is a precursor of glutathione and might be more efficiently absorbed in the intestine than glutathione [24,61]. Increasing the extracellular glutathione levels is not only beneficial for the host during phases of increased oxidative stress, but may also increase bacterial viability and survival during gastrointestinal passage [56].

Besides glutathione secretion, production of SCFAs such as acetate, propionate, and butyrate is also of central interest in the prevention of diseases and maintenance of gastrointestinal health. SCFAs are ingested through certain foods and produced by intestinal microbes. There is evidence that SCFAs have protective effects against the development of allergies, such as allergic rhinitis, asthma, and food allergies [62].

Probiotics may also play an important role in the improvement in iron uptake and thereby support adequate iron levels. Anaemia resulting from low iron levels is one of the most common nutritional disorders, mostly affecting infants and women [63]. Dietary iron leaves the stomach as Fe(III) and is reduced by ferrireductase (DcytB) in the duodenum to Fe(II), which is subsequently transferred across the apical membrane by divalent metal transporters. While several studies claim facilitation of iron absorption by probiotics, the mechanism is not yet clear. It was proposed that a reduced pH caused by lactic acid increases iron solubility and therefore absorption [64]. Furthermore, *L. fermentum* CECT5716 secretes p-hydroxyphenyllactic acid (HPLA). In vitro, HPLA from *L. fermentum* CECT5716 can reduce Fe(III) to Fe(II) and thereby renders it to the essential form that can be taken up by enterocytes [33]. HPLA is also discussed to have antifungal and biopreservative capacities [65]. This intriguing observation aligns with the observation of a low-molecular-weight fraction in HM with iron-reducing capacity, which enhances the iron absorption in newborns [66].

### 3.7. Postbiotic Mechanisms

The presence of viable bacteria is crucial for some of the beneficial effects of probiotics. However, bacterial factors derived from non-active microorganisms can also be beneficial. For instance, heat-inactivated *L. fermentum* CECT5716 was demonstrated to reduce inflammation caused by the induction of colitis in a rat model of gut inflammation [15]. Several studies in animals and humans have shown that some strains show health-promoting effects independent of their viability or even cell integrity. These effects may be due to the fact that host cells recognise bacterial motifs (e.g., DNA, cell wall components such as peptidoglycan, or lipopolysaccharides) via Toll-like receptors, which triggers an immunomodulatory effect [67]. These effects have come to be called “postbiotic”, though a recently published definition of “postbiotic” not only includes inanimate cells but also their produced metabolites [68]. By this definition, postbiotic effects have clearly been demonstrated for *L. fermentum* CECT5716. Postbiotics could be an alternative to probiotics for vulnerable individuals, for whom live probiotics might raise potential safety concerns.

## 4. Safety of *L. fermentum* CECT5716

*L. fermentum* CECT5716 is included in the list of taxonomic units proposed by the European Food Safety Authority (EFSA) for the qualified presumption as safe (QPS) status and is generally recognised as safe (GRAS Notification in 2015) by the US Food and Drug administration (FDA) [69]. However, despite their safe use over a long time and the general assumption of their safety, lactobacilli may theoretically and in rare cases cause negative side effects. Therefore, safety assessments regarding antibiotic susceptibility and virulence properties are recommended. When judging safety of new probiotic strains, criteria such as human origin, long history of safe usage, or absence of pathogenic traits are applied and therefore also tested for probiotic candidates [70,71]. The EFSA has created cut-off values for antibiotic susceptibility for probiotics [72]. Two independent studies have shown that the pattern of susceptibility of *L. fermentum* CECT5716 met the thresholds referenced by the EFSA, for all antibiotics tested [24,70]. Similarly, based on a systematic review, the European Society for Paediatric Gastroenterology Hepatology and Nutrition (ESPGHAN) committee concluded that probiotic-supplemented formula does not raise safety concerns [73].

Regarding toxicity, even at a concentration 10,000 times higher (expressed per kg of body weight) than those consumed normally by humans, no death, illness, or sign of negative activity was observed in a mouse model. Additionally, no translocation of *L. fermentum* CECT5716 to blood, liver or spleen was detected [70].

Another potential safety concern is the generation of biogenic amines such as histamine and tyramine, which can be generated by some microorganisms through the activity of amino acid decarboxylase. Ingestion of high amounts of biogenic amines such as histamine and tyramine can be mistaken for allergic reactions due to the similarity of the symptoms, e.g., facial flushing, sweating, rash, diarrhoea, and cramps [74]. However, the production of unwanted biogenic amines (tyramine, histamine, putrescine, and cadaverine) was not detected for *L. fermentum* CECT5716. Further, the production of prophages (i.e., bacteriophage genomes integrated into the host genome), which is of interest due to their involvement in the transduction of antibiotic resistance genes, could not be identified [23,24,75].

In addition to testing these safety properties in vitro and in vivo, several clinical studies in infants have confirmed the safety of *L. fermentum* CECT5716 for human use. The details of these randomised trials are further described in Section 6. For instance, a randomised study of 137 infants aged 1 month has shown that the growth of infants who had received either *L. fermentum* CECT5716-containing formula or the control formula was identical in both groups after 4 months and 6 months. The supplemented infants did not experience any bacterial complication relating to probiotic supplementation [76]. In a 3-year follow-up of this study comparing children who had received either *L. fermentum* CECT5716-containing formula or the control formula, no differences in growth or incidence of diseases were observed. Thus, the long-term safety of *L. fermentum* CECT5716 was confirmed [77].

In addition, preclinical studies can provide insight into the probiotic mechanisms of *L. fermentum* CECT5716 and thus inform on potential safety concerns, as described in the next section.

## 5. Preclinical Studies

A plethora of probiotic strains are assigned to putative health-promoting effects. Preclinical trials in several animal models of different pathologies have given first insights into mechanisms of action, which is an essential step in determining host- and pathology-tailored probiotics. Currently available animal-free testing models lack the complex interplay of different organ systems as well as the behaviour of the respective strain in the GIT. Nonetheless, the usage of animal models in testing probiotic strains should be strictly controlled and the benefit defended in front of an ethics committee to limit animal numbers. A summary of preclinical studies using *L. fermentum* CECT5716 is presented in Table 1.

### 5.1. Gastrointestinal Inflammation

Breastfeeding seems to prevent the development of inflammatory bowel diseases (IBD) such as Crohn´s disease or Ulcerative colitis in later life [87,88]. This effect might be mediated by the HMM. Several murine models for human IBD are available. They vary not only in their way of inducing the intestinal inflammation, but also in the type of immune response, location of inflammation, and affected structures [89]. Therefore, probiotic strains should be tested in different models to verify an IBD-protective capacity.

In a rat model of chemically induced colitis (trinitrobenzenesulfonic acid (TNBS)), the efficacy of various probiotics, including *L. fermentum* CECT5716, to prevent histological signs of inflammation was tested. The study showed that different probiotics exerted different mechanisms, ultimately resulting in a reduction in histological damage. While both *L. fermentum* CECT5716 and *L. reuteri* ATCC55730 prevented histological signs of inflammation and reduced the level of colonic myeloperoxidase infiltration (as marker of neutrophil infiltration), only *L. fermentum* CECT5716 significantly counteracted the colonic glutathione depletion induced by inflammatory processes. Further, a decrease in cyclooxygenase expression and an increase in SCFAs was only observed for *L. fermentum* CECT5716, a trait already described and associated with protection against exacerbated inflammatory processes [55,61].

In another chemically induced murine colitis model (dextrane sulphate sodium (DSS)) *L. fermentum* CECT5716 also reduced colonic histopathology. One possible mechanism of protection might be the preservation of intestinal barrier function. *L. fermentum* CECT5716 treatment sustained the expression of the barrier proteins occludin and ZO-1, which resulted in reduced intestinal permeability [42]. In another mouse study, *L. fermentum* CECT5716 attenuated TNBS-induced colitis, even when given in a therapeutic manner, i.e., after induction of pathology [79].

Although these are artificial models, they can help to elucidate probiotic mechanisms. For instance, *L. fermentum* CECT5716 treatment resulted in amelioration of the inflammatory response by counteracting the colonic glutathione depletion [61]. This antioxidant effect may be especially important in this model of gut inflammation, as TNBS administration is assumed to induce acute colonic injury mainly by the production of highly reactive oxygen species (ROS) in the early phase of colonic damage [79]. Another possible mechanism might be the *L. fermentum* CECT5716-mediated increase in luminal SCFA levels. SCFAs are serving as the main energy substrate for intestinal epithelial cells and ameliorate the IBD-related excessive immune response, by promoting regulatory T-cells [55,90,91].

Irritable bowel syndrome (IBS) is a widespread intestinal disorder, characterised by abdominal discomfort and pain, changed bowel habits, flatulence, and bloating. The aetiology is still unknown, but differences in the intestinal epithelial barrier function, composition of the gut microbiota, and immune response are discussed as possible pathophysiological mechanisms [92,93,94,95,96]. In a model of experimental IBS induced by deoxycholic acid (DCA) in rats, attenuation of visceral hypersensitivity, changes in gut inflammatory markers, and improvement of the intestinal barrier in experimental IBS were demonstrated. In behavioural studies, *L. fermentum* CECT5716 also improved anxiety-like behaviour, associated with experimental IBS, showing its potential to improve psychological stress [78].

### 5.2. Psychological Stress

Psychological stress, e.g., by maternal separation, can induce a profound stress response in infants that could impair the intestinal barrier function. This increased stress response can alter systemic cortisol levels and affect learning processes. Moreover, a stress-induced decrease in intestinal barrier function might be associated with long-term health consequences such as increased incidence of allergies [97,98]. A potential option to ameliorate these stress-induced consequences might be early life administration of probiotics, including *L. fermentum* CECT5716. A recent animal study investigated this hypothesis by exposing rat pups to psychological stress by maternal separation and water avoidance testing. In both tests, pups receiving *L. fermentum* CECT5716 showed improved intestinal barrier tightness and reduction in intestinal permeability (i.e., increased ZO-1 expression and decreased permeability to fluorescein sulfonic acid) in a rat model of maternal separation and water avoidance. Interestingly, ex vivo analysis showed spatial differences in the barrier protection effects. *L. fermentum* CECT5716 especially improved the ileal barrier, while having almost no effect on the proximal colon. Furthermore, they tested the probiotic effects on anxiety-like behaviour, ethological parameters, and locomotor activity in a maze setting. While anxiety-like behaviour did not change, the *L. fermentum* CECT5716-treated group showed increased exploratory behaviour during the test phase. While behavioural observations are subjective measures, plasma markers of barrier function (sulfonic acid) or stress (corticosterone) were improved by the probiotic treatment as well. The authors therefore concluded that *L. fermentum* CECT5716 may be a novel method for prevention and treatment of gastrointestinal disorders associated with reduced intestinal barrier function [35].

### 5.3. Hypertension

Hypertension is one of the main risk factors for cardiovascular events such as myocardial infarct or stroke. Current guidelines for the management of hypertension include dietary interventions with the aim to reduce the number and dosage of necessary medication. As the consumption of fermented milk products historically showed antihypertensive potential, probiotics might play a role in the reduction in hypertension. One proposed mechanism for this effect is the strengthening of the anti-inflammatory capacity, as endothelial inflammation may play an important role in causing endothelial dysfunction [99]. By using a spontaneously hypertensive rat (SHR) model (characterised by elevated blood pressure, arterial remodelling, endothelial dysfunction, vascular inflammation, and dysregulated immune system), orally administered *L. fermentum* CECT5716 reduced the systolic blood pressure. Along with this, an increased relaxant response was observed in the probiotic groups (as measured by the contractile responses). One of the key mechanisms of endothelial dysfunction is vascular generation of ROS, which is abolished via indirect effects by orally administered *L. fermentum* CECT5716. Further, the proinflammatory response in the aorta of these rats was reduced, as shown by attenuated tumour necrosis factor alpha (TNF-α) expression [80]. In the same animal model, *L. fermentum* CECT5716 was demonstrated to prevent the development of hypertension. Furthermore, an increase in butyrate-producing bacteria, improved balance of T-helper and T-regulatory cells, normalised endotoxaemia, and improved endothelial function were observed [81].

On the other hand, *L. fermentum* CECT5716 was not effective in a rat model of induced hypertension via administration of an inhibitor of nitric oxide synthase (NOS)-induced dysbiosis, endothelial inflammation, and hypertension [31]. However, the early signs in the atherosclerotic process, i.e., dysbiosis (in the respective work defined as increased Firmicutes/Bacteroidetes ratio), vascular oxidative stress, and inflammation (defined by aortic ROS accumulation and Th17 infiltration, respectively) could be attenuated by *L. fermentum* CECT5716 [31].

In a mouse model of induced hypertension, *L. fermentum* CECT5716 inhibited the development of hypertension and endothelial dysfunction, which were induced by tacrolimus. Further, the balance between T-helper and T-regulatory cells was restored. The authors hypothesised that these changes were mediated by the prevention of gut dysbiosis [82].

Systemic lupus erythematosus is an autoimmune disorder leading to multiple complications, including endothelial dysfunction and hypertension. In a mouse model of systemic lupus erythematosus, *L. fermentum* CECT5716 and/or *Bifidobacterium breve* CECT7263 were administered. Both probiotics prevented hypertension and endothelial dysfunction, reduced the plasma levels of autoantibodies against double-stranded DNA, activated Toll-like receptor 9 (TLR-9) expression, and reduced T-cell activation [83].

Taken together, these results show that although probiotics cannot be the sole treatment for hypertension, certain pathophysiological mechanisms can benefit from probiotic intervention.

### 5.4. Immunity

*L. fermentum* CECT5716 displayed beneficial effects on immunity when administered during pregnancy and lactation in rats. In lactating dams, a reduction in T-cytotoxic cells, modulation of intestinal cytokines, and change in fatty acid composition were observed, while their pups exhibited changes in immunoglobulins and fatty acid profile [85].

These results indicate that *L. fermentum* CECT5716 has beneficial effects on the immune system of mother and offspring.

Among hospitalised patients in intensive care units, sepsis is one of the most common causes of death. In 2016, the Sepsis-3 conference defined sepsis as a “life-threatening organ dysfunction caused by a dysregulated host response to infection” and septic shock as a “subset of sepsis in which circulatory, cellular, and metabolic abnormalities are associated with a greater risk of mortality” [100]. Sepsis is often a secondary complication, e.g., in patients suffering from trauma characterised by a reduction in cellular immune response. An initial event is the release of lipopolysaccharides (LPS) from bacteria into the bloodstream, shown also by the fact that septic shock can be induced by intraperitoneal application of LPS in animals. Interestingly, mice pre-treated with *L. fermentum* CECT5716 had significantly reduced LPS-induced changes in organ weight and improved biochemical parameters such as TNF-α levels after LPS injection, compared to the control group. The LPS-induced alteration in liver function, as evidenced by the decrease in glutathione levels, was not as marked in *L. fermentum* CECT5716-pre-treated mice as in the control group, a mechanism that could attenuate the deleterious effect of ROS often observed in sepsis [84].

### 5.5. Metabolic Disease

Metabolic syndrome is characterised by obesity, insulin resistance, fatty acid profile alterations, and hypertension. Likewise, emerging research suggests that the intestinal microbiota can have a large impact on the emergence of these conditions. Therefore, probiotic interventions could be an attractive therapy in the management of metabolic syndrome. For example, the effect of *L. fermentum* CECT5716 on metabolic syndrome was analysed in a preclinical study on rats that were fed a high-fructose diet, resulting in insulin resistance, liver steatosis, altered production of SCFAs, and changes in microbiota. Feeding a synbiotic containing *L. fermentum* CECT5716 and fructooligosaccharides ameliorated the effects of a high-fructose diet on liver steatosis, systemic inflammation, dysbiosis, and barrier function [45]. Similarly, beneficial effects were observed in a study on minipigs. Piglets were fed a probiotic formula with plant lipids, dairy lipids, and *L. fermentum* CECT5716, before being challenged with a high-energy diet. The probiotic formula had multiple beneficial effects, including modulation of gut microbiota, increase in SCFA concentration, and enhanced GLP-1 secretion [43]. These results indicate that *L. fermentum* CECT5716 supplementation in infants may decelerate or prevent development of metabolic diseases later in life.

### 5.6. Asthma and Allergy

Asthma is a bronchial inflammatory disease, causing shortness of breath, chest tightness, and other symptoms, thus presenting a significant impact on the quality of life of asthma patients. A recent study in ovalbumin-induced mice showed that supplementation with *L. fermentum* CECT5716 decreased the content of inflammatory factors in bronchoalveolar fluid and expression of TLR2/TLR4 in the duodenum and lungs. Further, inflammatory cell infiltration in the airway mucosa was reduced [86]. These results suggest that *L. fermentum* CECT5716 might be a novel complementary treatment for asthma, although further studies, especially human trials, are needed.

First ex vivo results with the serum of cow’s milk-allergic infants showed that *L. fermentum* CECT5716 did not show any allergenic potential when tested against specific antibodies (α-S1-casein, α-S2-casein, α-β-casein, α-κ-casein, α-lactalbumin, α-β-lactoglobulin, α-lactoferrin). In the same study, it was shown that in vitro, in combination with a galactooligosaccharide containing extensively hydrolysed infant formula, *L. fermentum* CECT5716 significantly reduced the allergenic activity of the infant formula. This was shown by a decreased basophil degranulation of rat basophil leukaemia (RBL) cells that express the human high-affinity receptor for immunoglobulin E (FcεRI) and were loaded with serum immunoglobulin E (IgE) from cow’s milk-allergic patients. These results suggest that *L. fermentum* CECT5716 might be a promising ingredient for hypoallergenic infant formulae [101].

Although preclinical studies have provided convincing evidence for the efficacy of *L. fermentum* CECT5716 to modulate host responses, specifically those relating to immune and gastrointestinal functions, translating and confirming these findings into clinical populations is of utmost importance. Thus, the following section summarises clinical studies on the efficacy of *L. fermentum* CECT5716 in humans.

## 6. Clinical Trials

The first years of human life are a period with a particularly high rate of infections. As explained previously, a relationship between immunity and the microbiota has been established in various studies [37,51,52]. Thus, it is hypothesised that support of the microbiota in early life, e.g., by breastfeeding, might prevent infections in infants and adults.

We have recently published the results of a randomised controlled clinical trial investigating the effect of *L. fermentum* CECT5716 on the infant microbiota during the first year of life. A total of 540 infants were included in the study and received, for a duration of 12 months, either the synbiotic intervention formula *L. fermentum* CECT5716 and galactooligosaccharides, or a standard formula without pro- or prebiotics. A non-randomised breastfed group was also included. At the age of 4 months, significantly higher prevalence and abundance of *Bifidobacterium* spp. and *Lactobacillaceae* in infants fed with formula supplemented with *L. fermentum* CECT5716 and galactooligosaccharides were demonstrated. The phylogenetic profiles of these infants were closer to the profiles of breastfed infants, compared to infants fed with the control formula [102].

Other randomised clinical studies have previously been conducted to investigate the safety and efficacy of *L. fermentum* CECT5716 in preventing infections. A summary of clinical trials in adults is presented in Table 2, and a summary of clinical trials in infants is presented in Table 3.

### 6.1. Mastitis

Mastitis is a breast infection that commonly occurs during lactation. The effects of oral administration of *L. fermentum* CECT5716 were studied in several groups of women with mastitis. In the study by Arroyo et al., 352 women with mastitis were either treated with *L. fermentum* CECT5716, *L. salivarius* CECT5713 (9 × 10^10^ log cfu/day, respectively), or antibiotics for a duration of 21 days. Probiotic supplementation was even more effective than antibiotic treatment in lowering bacterial count (*Staphylococcus epidermidis*, *Staphylococcus aureus*, and *Staphylococcus mitis*) in HM. In addition, patients treated with probiotics experienced a higher reduction in pain scores and lower recurrence of mastitis [30].

Two further studies also showed a significant reduction in *Staphylococcus* spp. in HM of mothers having received perinatal antibiotics or self-reported pain during breastfeeding. Here, mothers were treated with *L. fermentum* CECT5716 compared to a placebo [103,104]. In women reporting painful breastfeeding, a decrease in pain score was observed after a three-week supplementation with different doses of *L. fermentum* CECT5716 (3, 6, or 9 × 10^9^ cfu/day, respectively) [104]. Hereby, significantly less pain was observed after 7 days already. When *L. fermentum* CECT5716 was given in a preventative manner (3 × 10^9^ cfu/day), a 51% lower incidence of mastitis was observed during the four-month observation period [103].

A recent randomised controlled trial analysed the long-term impact of *L. fermentum* CECT5716 in women with breast abscesses. Three months after the trial, patients were followed up via an online questionnaire. There was no significant difference in the overall rate of stopping breastfeeding, pain relief, or recurrence of mastitis. However, a statistically significant difference in the rate of stopping breastfeeding due to the recurrence of mastitis was observed in the treatment group compared to the placebo group, showing that the supplementation of nursing women with *L. fermentum* CECT5716 might support exclusive breastfeeding [105].

### 6.2. Influenza Vaccination

Since influenza presents a large economic burden, controlling yearly influenza waves is of utmost importance. Therefore, vaccination is recommended. Since it was previously demonstrated that lactobacilli can improve immunity, the effect of *L. fermentum* CECT5716 on the immune response following influenza vaccination was analysed in healthy adults. In the study, 50 healthy adults were vaccinated and received either 1 × 10^10^ log cfu/day *L. fermentum* CECT5716 or placebo 2 weeks before and 2 weeks after vaccination. The probiotic group showed a significant increase in NK cells and helper and cytotoxic T-cells, compared to the placebo group. The production of antigen-specific IgA was also significantly enhanced [50]. Thus, probiotic supplementation could be an interesting approach to improving vaccine efficacy by promoting activation of the immune system.

### 6.3. Infections in Infants

Due to the high rate of infections, the first years of life are favourable for microbiota modification using probiotics. Thus, several clinical studies have been conducted to investigate whether *L. fermentum* CECT5716 can prevent infection in infants. Hereby, infants were either directly (via infant formula) or indirectly (via intake of probiotic capsules by breastfeeding mothers) supplemented with *L. fermentum* CECT5716. Interestingly, even the indirect supplementation resulted in the first compelling protective effects: for instance, a 16-week supplementation with *L. fermentum* CECT5716 in breastfeeding mothers after delivery led to a significantly reduced incidence of conjunctivitis in their infants [107].

Various studies concluded that no relevant changes in infant growth could be observed after supplementing infants with *L. fermentum* CECT5716 [76,77,106]. Further, the consumption of a formula containing *L. fermentum* CECT5716 was similar to that of the control formula. Importantly, tolerance and compliance were comparable between formula groups [76]. Thus, clinical trials confirm the safety and tolerability of *L. fermentum* CECT5716.

#### 6.3.1. Respiratory Tract Infections

Respiratory tract infections are among the most common diseases in infants. In fact, a recent study revealed that over 80% of infants had at least one upper respiratory tract infection during the first year of life [106]. Breastfeeding is known to decrease the incidence of upper and lower respiratory tract infections, which may involve complementary immunological mechanisms of action [108,109].

Similarly, it was demonstrated that the *L. fermentum* CECT5716-supplemented formula can reduce the incidence of upper respiratory tract infections by 27% in infants aged 6 to 12 months (the incidence rate during the study period: in the probiotic group, 0.969 ± 0.96; in the control group, 1.330 ± 1.23) [32]. This was not confirmed in a different formula trial using *L. fermentum* CECT5716 in infants aged 1 to 6 months, where no significant difference in incidence of respiratory tract infections was observed [76], probably due to the low incidence of respiratory tract infections in this age group. Another formula study reported no general difference in incidence of respiratory tract infections, although a subgroup analysis of infants born by caesarean section revealed a reduction in upper respiratory tract infections after *L. fermentum* CECT5716 administration from 1 to 12 months of age [106].

#### 6.3.2. Gastroenteritis

Diarrhoea caused by gastroenteritis is a mostly viral infection affecting predominantly infants and young children. The prevalence in young children (<3 years) is 0.5 to 2 episodes per child [110]. These infections are widespread throughout the year but usually spread in the form of winter epidemics, and they represent a frequent cause of mortality in developing countries, leading to high health and social costs in industrialised areas.

Breastfeeding is the most effective way of reducing the incidence and severity of these infections in infants. One mechanism through which breastfeeding can prevent infections is fostering the development of the infant microbiota. Thus, in addition to developing vaccines, modulating the microbiota of non-breastfed infants has been explored. Several probiotic strains have been shown to significantly reduce the incidence of gastroenteritis. *L. fermentum* CECT5716 administration for 5 months showed a significant (71%) reduction in gastroenteritis in infants aged 1 to 6 months (the incidence rate during the study period: in probiotic group, 0.082 ± 0.04; in the control group, 0.283 ± 0.07) [76]. In a second clinical trial, supplementing infants aged 6 to 12 months with *L. fermentum* CECT5716 led to a significant (46%) reduction in the incidence of gastrointestinal infections as well (the incidence rate during the study period: in the probiotic group, 0.196 ± 0.51; in control group, 0.363 ± 0.53) [32]. In both studies, infants in the probiotic as well as the control group received galactooligosaccharides as prebiotics. Furthermore, a third clinical study in infants aged 1 to 2 months concluded that *L. fermentum* CECT5716 decreased the incidence of diarrhoea by 44% and the duration by 2.5 days [106]. All three studies demonstrated good tolerability of the study formula enriched with *L. fermentum* CECT5716 [32,76,106].

Furthermore, these three studies were analysed in a recent systematic review and meta-analysis. This review concluded that *L. fermentum* CECT5716 at doses from 1 × 10^9^ to 8.4 × 10^8^ cfu/day in milk formulas led to a significantly reduced incidence of gastrointestinal infections in infants up to 12 months. The authors suggested that future long-term studies should be conducted [111]. In addition, the effects observed in these three study cohorts were specifically analysed for those infants delivered by caesarean section. The results revealed a statistically significant reduction (73%) in gastrointestinal infections in infants born by caesarean section who received *L. fermentum* CECT5716 [112]. As subgroup analyses of intervention studies in infants have shown stronger effects in infants born by caesarean section, further studies focussing on these infants will give valuable insights for this specific group. The microbiota of infants born by caesarean section might benefit even more from probiotic intervention, as their microbiota might have vacant niches in their early phase of life [102]. Therefore, a current study recruiting 486 infants (NCT04991792) is being conducted to assess the positive effect of feeding a formula containing *L. fermentum* CECT5716 and GOS on the composition of the gut microbiota in caesarean section (CS)-born healthy term infants within the first 6 months of life. Current and future studies will give further insights into the probiotic, as well as postbiotic mechanisms performed by *L. fermentum* CECT5716 in adults with a predisposition for certain diseases (e.g., IBD), in nursing mothers with a risk of mastitis development, as well as in infants who cannot be breastfed.

## 7. Conclusions

*L. fermentum* CECT5716 is a probiotic strain isolated from HM. Several preclinical studies have demonstrated the health-promoting effects of *L. fermentum* CECT5716 in models of gastrointestinal inflammation, psychological stress, hypertension, immunity, metabolic disease, and asthma. Its positive effect on the microbiota in a synbiotic formulation was shown in various clinical trials investigating mastitis, influenza vaccination, respiratory tract infections, and gastroenteritis. The ESPGHAN criteria for safety are met by *L. fermentum* CECT5716, and several clinical trials have confirmed the good tolerability and safety of an *L. fermentum* CECT5716-containing formula.

Future evaluations on the long-term health effects of *L. fermentum* CECT5716 may provide further insight into its use. Therefore, more intervention studies in infants with longer observation periods will give further insights into its health promoting effects. As probiotic effects might be modified by formula compounds, e.g., hydrolysed protein, prebiotics, or fats, studies with formulas differing in these compounds will be important, to specify certain probiotic or postbiotic mechanisms. Currently, several interventional studies are being performed to further support the existing results and explore new probiotic potentials of this strain.

## Figures and Tables

**Table 1:** Overview of preclinical studies for L. fermentum CECT5716.

Model Disease/Condition	Animal Model	Main Results	Reference
IBS induced by DCA	Rat	Reduction in signs of intestinal hypersensitivity, reduction in inflammation, and improvement in intestinal barrier integrity	Rodriguez-Sojo et al., 2022 [78]
Colitis induced by TNBS	Rat	Reduction in histological signs of inflammation	Peran et al., 2006 [61], Peran et al., 2007 [55]
Colitis induced by TNBS	Mouse	Reduction in histological signs of inflammation	Mane et al., 2009 [79]
Colitis induced by DSS	Mouse	Reduction in histological signs of inflammation	Rodriguez-Nogales et al., 2017 [42]
Maternal separation, Water avoidance stress	Rat	Prevention of stress-induced ZO-1 disorganization in epithelial cells and plasma hypercorticosteronaemia; increased exploratory behaviour	Vanhaecke et al., 2016 [35]
Hypertension (in SHR)	Rat	Reduction in vascular ROS generation, proinflammatory response, and systolic blood pressure	Gomez-Guzman et al., 2015 [80]
Hypertension (in SHR)	Rat	Prevention of hypertension; increase in butyrate-producing bacteria; improved balance of T-helper and T-regulatory cells	Robles-Vera et al., 2020 [81]
Hypertension induced by NO blockade	Rat	Reduction in dysbiosis, vascular oxidative stress, and inflammation	Robles-Vera et al., 2018 [31]
Hypertension induced by tacrolimus	Mouse	Prevention of hypertension and endothelial dysfunction; improved balance of T-helper and T-regulatory cells	Toral et al., 2018 [82]
Hypertension and systemic lupus erythematosus, induced by TLR-7 agonist	Mouse	Prevention of hypertension; reduction in autoantibodies; activation of TLR-9; reduced T-cell activation	Visitación et al., 2021 [83]
Septic shock induced by LPS	Mouse	Reduction in LPS-induced changes in organ weight, TNF-α levels, and liver function	Arribas et al., 2008 [84]
Immunity in pregnancy and lactation	Rat	Reduction in T-cytotoxic cells and modulation of intestinal cytokines and fatty acid profile; modulation of immunoglobulins and fatty acid profile in pups	Azagra-Boronat et al., 2020 [85]
Metabolic syndrome induced by HFD	Rat	Prevention of liver steatosis and systemic inflammation; amelioration of dysbiosis and barrier function (synbiotic treatment with *L. fermentum* CECT5716 + fructooligosaccharides)	Rivero-Gutiérrez et al., 2017 [45]
Overweight induced by HED	Pig	Increase in SCFAs; improvement in endocrine function, e.g., GLP-1 (treatment with *L. fermentum* CECT5716 + plant/dairy lipids)	Lemaire et al., 2018 [43]
Asthma induced by ovalbumin	Mouse	Reduction in inflammatory response and inflammatory cell infiltration in lung	Wang et al., 2022 [86]

DCA: deoxycholic acid; DSS: dextrane sulphate sodium; GLP-1: glucagon-like peptide 1; HED: high energy diet; HFD: high fructose diet; IBS: irritable bowel syndrome; LPS: lipopolysaccharides; NO: nitric oxide; ROS: reactive oxygen species; SCFA: short-chain fatty acids; SHR: spontaneously hypertensive rats; TLR: Toll-like receptor; TNBS: trinitrobenzenesulfonic acid; TNF-α: tumour necrosis factor alpha; ZO-1: zona occludens 1.

**Table 2:** Overview of clinical trials for oral administration of L. fermentum CECT5716 in adults.

Condition	Participants	Study Design	Main results	Reference
Mastitis	625 lactating mothers	Randomised, double blinded, controlled	51% reduction in the incidence rate of mastitis in the probiotic group	Hurtado et al., 2017 [103]
Mastitis	352 lactating mothers with mastitis	Randomised, controlled	Lower bacterial count in HM samples of probiotic group, compared to the control group	Arroyo et al., 2010 [30]
Mastitis	148 lactating mothers with breast pain	Randomised, double blinded, controlled	Significant decrease in bacterial load in HM samples of the probiotic group	Maldonado-Lobon et al., 2015 [104]
Mastitis	101 lactating mothers with breast abscess	Multicentre, randomised, double blinded, controlled	Significant decrease in the rate of stopping breastfeeding due to recurrence of mastitis	Zhang et al., 2022 [105]
Influenza vaccination	50 healthy adults (31 male, 19 female)	Randomised, double blinded, placebo controlled	Significantly lower incidence of influenza-like illness; significant increase in antigen-specific IgA in the probiotic group	Olivares et al., 2007 [50]

IgA: Immunoglobulin A, HM: Human milk.

**Table 3:** Overview of clinical trials for oral administration of L. fermentum CECT5716 in infants.

Condition	Participants	Study Design	Main Results	Reference
Gastrointestinal and upper respiratory tract infections	215 infants	Randomised, double blinded, controlled	Significant reduction in the incidence rate of gastrointestinal and upper respiratory tract infections in the probiotic group	Maldonado et al., 2012 [32]
Gastrointestinal infection and safety	137 infants	Randomised, double blinded, controlled	Significant reduction in gastrointestinal infection; normal growth and weight gain; normal consumption of formula; no symptoms relating to formula	Gil-Campos et al., 2012 [76]
Modulation of infant microbiota	540 infants	Randomised, double blinded, controlled, multicentre	Significant effects on microbiota states: phylogenetic profiles of the infants receiving synbiotic infant formula were closer to reference profiles of those fed with HM	Lagkouvardos et al., 2022 [102]
Infection and safety	236 infants	Randomised, double blinded, controlled	44% lower incidence of diarrhoea and 2.5 days reduction in duration of diarrhoea; lower incidence of respiratory tract infections among infants born by caesarean section; normal growth	Maldonado et al., 2019 [106]
Long-term safety	110 infants	3-year follow-up study	No significant difference in growth and incidence of infectious and non-infectious gastrointestinal diseases	Maldonado-Lobon et al., 2015 [77]
Infection and growth	625 mother–infant pairs	Randomised, double blinded, placebo controlled	Lower incidence of conjunctivitis in infants in the probiotic group; higher weight of infants in probiotic group	Pastor-Villaescusa et al., 2020 [107]

## Data Availability

Not applicable.
